# Accessing and navigating healthcare: A scoping review of the experiences of women of refugee background from Myanmar

**DOI:** 10.1111/hsc.13955

**Published:** 2022-08-01

**Authors:** Georgia Griffin, Mohammed Ali, S. Zaung Nau, Elisha Riggs, Jaya A. R. Dantas

**Affiliations:** ^1^ Curtin School of Population Health Curtin University Bentley Western Australia Australia; ^2^ School of Management and Marketing Curtin University Bentley Western Australia Australia; ^3^ Murdoch Children's Research Institute Parkville Victoria Australia; ^4^ Department of General Practice University of Melbourne Melbourne Victoria Australia

**Keywords:** Healthcare access, Intersectionality, Myanmar, Refugee women, Refugees, Survivorship

## Abstract

Despite well‐documented health problems, healthcare access by women of refugee background in resettlement countries is typically poor. Suggested reasons include inadequate health literacy and resettlement challenges. A scoping review to explore the experiences of women of refugee background from Myanmar accessing and navigating healthcare was conducted following Arksey and O′Malley's framework, with an intersectional lens. Studies were analysed thematically following Braun and Clark's approach; four themes (eight subthemes) were constructed: Culture (Constructions of health; Navigating cultural tensions); Gender (Shifting gender roles; Sexual and reproductive health); Survivorship (Past health experiences; Strength in collectivism); and Language (The language barrier; Masked communication barriers). Intersectional factors of culture, gender, survivorship and language influenced women's experiences, shaping barriers and facilitators to healthcare. Community networks and bicultural peers are resources which may be enhanced. Research into trauma‐informed cultural competency programs, community education and bicultural health navigators is recommended to support women of refugee background from Myanmar.

**What is known about this topic**

- Ongoing political unrest and violence are contributing to increasing numbers of refugees and displaced persons from Myanmar.
- Health services are underutilised by women in Myanmar and in resettlement countries.
- Limited health literacy can be a factor in the underutilisation of health services by people of migrant and refugee backgrounds.

**What this paper adds**

- Gender, culture, survivorship and language shaped barriers to accessing and navigating healthcare.
- Pre‐existing community capacity including bicultural peers and community networks may be drawn upon to co‐design health interventions and healthcare provider training.
- For women of refugee background from Myanmar, communication was impeded by language barriers, health literacy and self‐effacing tendencies.

## 1 INTRODUCTION

Globally Myanmar, previously known as Burma, is one of the top five countries of origin from which refugees flee (UNHCR, 2020). Decades of military dictatorship and human rights violations have resulted in over one million people being displaced or seeking asylum in neighbouring countries such as Thailand or Bangladesh (Borwick et al., 2013; Fike & Androff, 2016). Through the United Nations Human Rights Commission resettlement program, refugees may resettle in countries which have ratified the 1951 Refugee Convention or 1967 Protocol (Millbank, 2000). Since 2019, the most common signatory countries in which people from Myanmar have resettled are the United States of America, Australia, Canada, New Zealand and South Korea (UNHCR, n.d.). With the most recent wave of political unrest since February 1, 2021, the numbers of refugees and displaced persons from Myanmar are rapidly growing (UNHCR Regional Bureau for Asia and the Pacific, 2022). Currently, approximately one million people from Myanmar are living in neighbouring countries as refugees and asylum‐seekers, and an additional 873,000 people are internally displaced within Myanmar (UNHCR Regional Bureau for Asia and Pacific, 2022).

### 1.1 Health inequity

Countries that are signatories to the Refugee Convention have an obligation to meet the health needs of resettled refugees (Parajuli et al., 2020). For women of refugee background from Myanmar, these needs are often complex (Maung et al., 2021). In Myanmar, restricted healthcare access and conflict have resulted in high rates of mental health trauma, malnutrition and infectious diseases along with other morbidities and mortality in the general population (Hoffman & Robertson, 2016). Health services are underutilised by women in Myanmar (Htun et al., 2021; Milkowska‐Shibata et al., 2020). Furthermore, women experience significant health inequity through targeted physical and sexual violence, abduction and ethnic cleansing campaigns (Oo & Kusakabe, 2010).

In refugee camps in neighbouring countries such as Thailand, Malaysia and Bangladesh, this burden of disease and violence continues. Well‐documented health problems include severe malnutrition, mental illness, overcrowding and communicable and non‐communicable diseases (Parmar et al., 2019; Wali et al., 2018). Women can be vulnerable to sexual and gender‐based violence, early marriage and poor perinatal care (Jops et al., 2019; Parmar et al., 2019; Wali et al., 2018). Health services are limited and often difficult to access or utilise (Parmar et al., 2019). People seeking refuge from Myanmar may have lived for years, even decades, in these conditions with limited healthcare and limited opportunities for employment, uncertain of their futures and resettlement pathways (Fike & Androff, 2014). This protracted situation has implications for peoples' physical and mental health.

On arrival in resettlement countries, specific health and settlement services for people of refugee background facilitate their orientation to their new country and their healthcare access (Fike & Androff, 2016; Taylor & Lamaro Haintz, 2018; Tuteja et al., 2022). The scope and governance of these services vary depending on resettlement location and in some countries, by visa type (Kumar, 2021; Taylor & Lamaro Haintz, 2018). Settlement services are typically designed to target newly arrived refugee background populations (Renzaho et al., 2022). Given the complex and prolonged nature of their health needs, healthcare access and health outcomes remain poor following resettlement (Parajuli & Horey, 2020; Taylor & Lamaro Haintz, 2018).

Women of refugee background specifically experience poorer health outcomes than those not of refugee background due to impacts of war, trauma, language, flight and resettlement (Kentoffio et al., 2016). They may continue to face complex obstetric problems, poor perinatal outcomes, sexual violence and unwanted pregnancy (Heslehurst et al., 2018). Trauma and mismanaged health prior to resettlement may have ongoing physical and psychological consequences (Parajuli & Horey, 2020). Women face barriers to health service access due to limited education with associated implications for health literacy (Hawkins et al., 2021; Kentoffio et al., 2016). Additional reasons for poor healthcare access and outcomes include resettlement challenges, perceived restricted or unavailable healthcare access, and confusion about healthcare entitlements related to visa type (Taylor & Lamaro Haintz, 2018).

Health literacy is a key factor in migrant and refugee health outcomes (Becerra et al., 2015; Todd & Hoffman‐Goetz, 2011; Tsai & Lee, 2016). Health literacy refers to “the literacy and numeracy skills that enable individuals to obtain, understand, appraise, and use information to make decisions and take actions that will have an impact on health status” (Nutbeam & Lloyd, 2021, p161). Thus, health literacy is an essential component of an individual's ability to access and navigate appropriate healthcare services (Nutbeam & Lloyd, 2021). Where health literacy is limited, people of migrant and refugee backgrounds may be vulnerable to underutilisation of health services including preventative health services (Lee et al., 2015). When people do not engage with healthcare effectively, health conditions and general poor health are exacerbated (Parajuli & Horey, 2020).

As women of refugee background from Myanmar resettle in signatory countries such as Australia or the United States of America, they bring with them complex and diverse health needs. Health inequity is increasingly understood through an intersectional lens; that is factors such as gender and culture may influence the health service access and health outcomes of an individual or subpopulation (Couto et al., 2019). It is evident that women of refugee background from Myanmar may have experienced an array of threats to their health including those specific to their sex such as poor perinatal care or gender‐based violence (Jops et al., 2019; Parmar et al., 2019). While interventions exist to improve the health outcomes of people of refugee background and women specifically, an understanding of the intersectional factors which influence the women's health service access is required to ensure that these interventions may be effective for this subpopulation (Hawkins et al., 2021).

This scoping review aims to consolidate the existing international literature, offering insight into the specific experiences of women of refugee background from Myanmar accessing and navigating healthcare and the intersectional factors which may influence these experiences. Such insight is essential for health professionals and policymakers to support women of refugee background from Myanmar to engage with health services to improve health outcomes.

### 1.2 Theoretical framework ‐ Intersectionality

This scoping review is underpinned by intersectionality theory which posits that individuals are subject to intersecting social locations or factors that influence their experiences of discrimination or inequity (Crenshaw, 1989). An intersectional understanding of health allows for the complexity of interactions between these social factors to be identified and examined; understanding of the interplay between these factors is crucial to the design and implementation of future public health interventions for specific subpopulations or communities (Couto et al., 2019).

Intersectionality theory has traditionally identified class, gender, culture and race or ethnicity as important factors in health inequalities (Couto et al., 2019). Women of refugee background from Myanmar are a unique, ethnically diverse cohort. Myanmar is composed of numerous ethnic, linguistic and religious groups. These differences have been used to justify and direct persecution and conflict (Parmar et al., 2019). Intersectionality theory provides an avenue for exploring the complex overlapping factors experienced by women of refugee background (Bowleg, 2012; Guruge & Khanlou, 2004).

Intersectionality theory guided the design and objectives of this scoping review and the thematic analysis of the included articles. Individuals are recognised as the experts of their own experiences in intersectionality theory (Crenshaw, 1989). Hence, this scoping review maintained a focus on the women's experiences. During analysis, barriers and facilitators were considered in the context of the overlapping factors which influenced them. The findings presented offer insight into the intersectional factors which influence the women's experiences of accessing and navigating healthcare.

## 2 MATERIALS AND METHODS

We undertook a scoping review to examine the limited research published on the healthcare experiences of women of refugee background from Myanmar (Levac et al., 2010; Peters et al., 2020). The five‐stage framework proposed by Arksey and O'Malley (2005) was followed to conduct this review. These five stages are: (1) identifying the research question; (2) identifying relevant studies; (3) study selection; (4) charting the data and (5) collating, reporting and summarising the results (Arksey & O'Malley, 2005). Each stage is outlined below.

A preliminary search of the Cochrane Library, CINAHL, the Joanna Briggs Institute Database, the Open Science Framework, PROSPERO and Medline did not reveal any reviews on this topic. The protocol for this scoping review was published in the Open Science Framework (Griffin et al., 2022).

### 2.1 Stage 1: Identifying the research question

The scoping review aims to address the following question:

What are the experiences of women of refugee background from Myanmar accessing and navigating healthcare on resettlement?

Two objectives guided the review and analysis:

1. To identify facilitators and barriers to healthcare access and navigation for women of refugee background from Myanmar on resettlement, and
2. To identify and explore intersectional factors which influenced the women's experiences.

### 2.2 Stage 2: Identifying relevant studies

#### 2.2.1 Inclusion criteria

##### Participants

Literature was considered if it explored the experiences of women of refugee background from Myanmar. This included studies which were not specific to women of refugee background from Myanmar, however, women of refugee background from Myanmar were included in the study cohort and findings specific to them were identifiable.

##### Concept of Interest

Studies which explored women's experiences of health, ill health, healthcare or health services, traditional health practices and self‐management of health needs were considered.

##### Context

Eligible studies explored the healthcare experiences of women of refugee background from Myanmar on resettlement in signatory countries, that is, countries which ratified the 1951 Refugee Convention or 1967 Protocol. Studies were not restricted on the basis of length of resettlement or time since arrival to allow for the breadth of women's experiences to be captured. Studies specific to experiences in refugee camps, international border regions or countries of asylum were excluded.

##### Types of Sources

Quantitative, qualitative and mixed methods studies, published in English, were considered for inclusion. Intervention studies were excluded as they are designed to evaluate an intervention rather than report on women's unmodified experiences. Literature was not restricted by year of publication to enable a broad range of women's experiences to be included. The database search was conducted in February 2021.

#### 2.2.2 Search strategy

The University research librarian assisted in the development of a search strategy modelled on the three‐step approach proposed by Peters et al. (2020). According to this approach, (1) an initial search of Medline was conducted; (2) index terms and keywords were identified from the titles and abstracts identified in this initial search and (3) these index terms and keywords were then used to search the databases. The reference lists of included sources were also searched for further eligible sources (Peters et al., 2020). Five comprehensive health science databases, CINAHL, Medline, PsycInfo, SCOPUS and ProQuest, were searched. Mednar was searched for grey literature such as policy statements and reports by research, government or non‐government organisations. A sample of the Medline search strategy is presented in Table 1.

**TABLE 1:** Medline search strategy.

Search	Terms	Records retrieved 19 February 2021
1	Refugees	10,707
2	Transients and Migrants	12,092
3	(“asylum seeker*” or refugee* or migrant* or “displaced person*”)	38,223
4	1 or 2 or 3	38,223
5	Health Knowledge, Attitudes, Practice	118,828
6	Health experiences	688
7	Attitude to Health	84,330
8	(“health experience*” or “health information” or “health service*” or healthcare or “health care”)	1,301,215
9	5 or 6 or 7 or 8	1,432,010
10	Burma	2617
11	(Burma or Burmese or Rohingya* or Myanmar or Karen or Karenni or Kachin or Bamar or Burman or Shan or Mon or Rakhine or Arakanese or Chin or Kayah)	21,297
12	10 or 11	21,297
13	4 and 9 and 12	213

### 2.3 Stage 3: Study selection

Titles and abstracts were screened by the first author (GG) to identify potential sources for inclusion. Two authors (JARD, MA or GG) independently reviewed the full text of each article against the inclusion criteria. Where there were discrepancies between two authors, the third author was consulted. The article was discussed until consensus was achieved. Each source identified for inclusion was assessed for rigour against the appropriate Critical Appraisal Skills Programme checklist (Critical Appraisal Skills Programme, 2019). No studies needed to be excluded on the basis of rigour.

**FIGURE 1:**
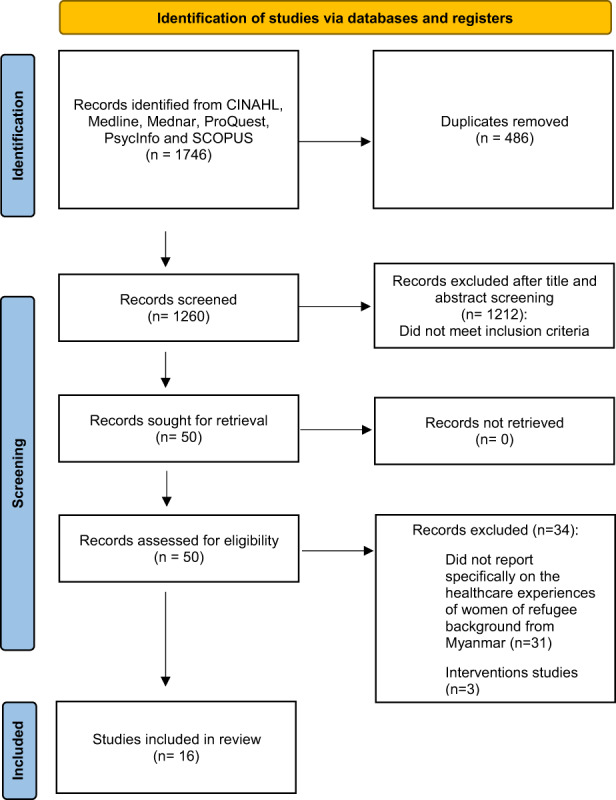
PRISMA flow diagram, modified from Page et al. (2021).

The initial database search retrieved 1746 records. After the removal of duplicates, 1260 articles underwent title and abstract screening. Following screening, 50 articles were sourced for full‐text review. Further studies were excluded because they were intervention studies (*n* = 3) or did not specifically explore the healthcare experiences of women of refugee background from Myanmar amongst larger cohorts (*n* = 31). Figure 1 shows a PRISMA flowchart illustrating the search strategy process.

### 2.4 Stage 4: Charting the data

Data were extracted from each included study using a table based on the template proposed by Peters et al. (2020). Extracted data included authors, year of publication, resettlement country, participants, purpose, methodology including the involvement of bicultural research team members, barriers to and facilitators of healthcare access or navigation. Table 2 presents the extracted data in categories aligning with the first review objective.

**TABLE 2:** Included studies and relevant findings

Authors (Year)	Country	Participants	Purpose	Methodology (Bicultural researchers)	Barriers to Healthcare Access	Facilitators of Healthcare Access
Clark (2018)	Canada	12 Karen refugee women 26 community health and social service providers	To describe Karen refugee women's experience of resettlement and the social structural factors which facilitated and/or challenged community capacity to support their mental health and well‐being during resettlement	Qualitative Ethnographic (Community leaders recruited participants; English proficient Karen women translated for participants with low English proficiency)	Language (lack of interpreters or resources); Resettlement stress and worry; Cultural vulnerability and stigma; Gendered responsibilities of childcare; Lower literacy; Health literacy; Service providers' lack of knowledge of or preparedness to care for Karen women	Utilising trusted settlement workers and interpreters; Offering longer medical appointments; Practicing cultural safety; Collaborating across services; Building capacity within Karen community; Becoming interpreters for own community
Jiwrajka et al. (2017)	Australia	1 Rohingya refugee woman	To follow the medical journey of the woman and explore the factors that impeded her care	Case Report	Dependant on husband for transport; Translator spoke wrong dialect; Not provided written information in any language; Doctors not trained to use telephone interpreter; Time constraints; Family members as translators; Limited experience with medications; Misalignment of doctor and patient goals	Universal healthcare scheme (Medicare)
Joseph et al. (2019)	Australia	Refugee mothers from Myanmar: 12 Karen 3 Karenni 4 Chin 3 Kachin 16 refugee mothers from Vietnam	To explore how postpartum support networks, hospital stay and healthcare services had an impact on breastfeeding experiences of refugee women from Vietnam and Myanmar	Qualitative (Bicultural workers facilitated recruitment, interviews, translation and transcription)		Observing traditional practices associated with health
Joseph et al. (2020)	Australia	Refugee mothers from Myanmar: 12 Karen 3 Karenni 4 Chin 3 Kachin 16 refugee mothers from Vietnam	To explore the infant feeding perspectives of refugees from Vietnam and four ethnic Myanmarese minorities by focusing on traditional perceptions related to infant feeding	Qualitative Postmodern inquiry (Bicultural workers facilitated recruitment, interviews, translation and transcription)		Drawing upon support from networks established in transit countries; Adapting or stopping some traditions deemed no longer necessary
Kim et al. (2021)	USA	11 Burmese community leaders: 7 Karen 3 Burman 1 Other ethnicity	To explore perceptions of mental illnesses and barriers to mental health service use as well as solutions to current mental health problems	Qualitative	Lack of understanding about mental health; Language barriers; Cultural stigma; Alternative treatments; Unresponsive system	Community education; Culturally‐competent providers; Care beyond mental health treatment
Kumar (2021)	USA	Burmese community members Healthcare providers NGO caseworkers	To explore the narratives of Burmese refugees in the United States through in‐depth interviews focusing on their meaning‐making practices surrounding health and displays of agency in the face of structural healthcare barriers	Qualitative (Interpreters for some interviews)	Employment‐insurance nexus; Different cultural articulations of health; Language barriers (Inability to communicate, making appointments, arranging transport, meeting government requirements); Inexperience with structural access to healthcare, including primary healthcare	Utilising community networks (e.g. Churches); Making appointments in person while already at health service; Seeking out providers with interpreters on recommendations from community; Community leaders distributing health information by email
LaMancuso et al. (2016)	USA	14 Karen refugee women 8 Karen interpreters/ doulas and key informants 6 medical providers	To describe Karen women's perinatal experiences and the role of doulas/interpreters in facilitating patient‐provider communication	Qualitative (Interpreters facilitated recruitment, development of interview guide, interviews)	Transportation; Hospital navigation; Language barriers; History of trauma; Depression; Domestic violence; Perceived agreeability/shyness and reluctance to ask questions	Having a doula; Training to become a doula
Lenderts et al. (2021)	USA	12 Karen refugee women	To explore how a sample of resettled Karen refugee women construct meaning around health, particularly in the context of cultural values, community and migration	Qualitative Ethnographic (Karen interpreter facilitated scheduling, follow‐up, interviews)		Asking each other for help, resources and guidance; Asking a friend to translate or make appointments
Lor et al. (2018)	USA	31 Burmese refugee women 27 Bhutanese refugee women	To gather information about factors influencing cervical cancer screening	Qualitative (Female Karen‐speaking focus group moderator from local community facilitated recruitment, focus groups)	Limited English proficiency; Problems with interpreters; Financial concerns; Transportation; Difficulty navigating the healthcare system; Embarrassment and stigma	Service providers forming positive relationships with women; Seeking information from family and friends; Retaining confidence to make own decisions
Maung et al. (2021)	USA	11 refugee women from Myanmar: 8 Karen 3 Bamar	To examine the strengths and posttraumatic growth experiences of a community of female refugees from Myanmar resettled in a Midwestern city in the United States	Qualitative (One researcher of Chinese‐Burmese heritage; key community informants facilitated development of interview guide, recruitment; translators assisted with Interviews)		Mentoring others; Sharing information between family and community members
Niner et al. (2013)	Australia	8 Karen refugee women	To understand how pregnancy and birth was experienced both before and after resettlement in Australia by forced migrant women	Qualitative Ethnographic (Female bilingual refugee background Karen research assistant facilitated interviews, translation)	Poor communication	Having confidence to question healthcare professionals and care
Niner et al. (2014)	Australia	15 Karen refugee mothers	To explore the ways in which displaced Karen mothers expressed emotions in narrative accounts of motherhood and displacement	Qualitative Ethnographic (Female refugee background Karen co‐researcher and cultural informant)	Translational challenges	Sharing information from pregnant colleagues
Riggs et al. (2012)	Australia	87 refugee mothers: 47 Karen Burmese 18 Iraqi Assyrian Chaldean 8 Assyrian Chaldean Lebanese 7 South Sudanese 7 Bhutanese 18 service providers	To explore experiences of using maternal and child health services (MCH), from the perspective of families from refugee backgrounds and service providers	Qualitative (Known bilingual worker or health worker facilitated recruitment; bilingual community workers and accredited interpreters contributed to development of focus group guides, focus groups)	Limitations in referral process; Access to transportation; Lack of confidence or ability to speak English; Making telephone bookings; Perceived lack of access to translated health information	Caseworker referral process; Refugee mentor linked to MCH nurse; Group appointments for parents attending playgroups; Continuity of nurse; Interpreter or bilingual staff
Schuster et al. (2019)	USA	15 Karen refugees (87% women) 15 Somali Bantu refugees (87% women)	To characterise Somali Bantu and Karen experiences with cancer and cancer screenings prior to and subsequent to resettlement in Buffalo, NY in order to inform engagement by health providers	Qualitative Community‐based Participatory Research (Community consultants facilitated recruitment, reviewed interview guides, interviews)	Transportation; Communication barriers; Perceived ineffectiveness of treatment and resultant mortality; Unfamiliarity with preventative medicine; Unfamiliarlocations; Lack of health insurance; Scheduling screening; Competing time demands	Interpretation; Eligibility for health insurance
Soin et al. (2020)	USA	32 refugee women: 12 from Myanmar 10 from Bhutan/Nepal 10 from Iraq	To learn about family planning practices of the resettled refugees and the factors that promote or hinder contraceptive use; and to understand how to effectively counsel and provide culturally sensitive family planning care to resettled refugee women	Qualitative (Bilingual community navigators and female translators facilitated recruitment, focus groups and interviews)	Young age; Lack of education; Differences in culture and language; Embarrassment	Improved access to information on resettlement; Health provider initiating conversation about family planning; Willingness to diverge from cultural norms; Informing each other about contraceptives
Ussher et al. (2012)	Australia	42 refugee women: 26 Karen 16 Assyrian	To examine constructions and experiences of reproductive and sexual health and associated services in two cultural groups whose experiences have not previously been examined: Assyrian and Karen women from Western Sydney, who arrived in Australia as refugees	Qualitative (Key community informants advised on research methods and questions; professional interpreter assisted focus group discussions)	Cultural taboos around sex and sexuality; Sexual health services not tailored to this population; Embarrassment; High cultural value of motherhood	High cultural value of motherhood; Education in refugee camps prior to resettlement

### 2.5 Stage 5: Collating, summarising and reporting the results

Two authors (JARD, MA or GG) independently conducted thematic analysis on each study, following Braun and Clarke's six‐phase methodology (Braun & Clarke, 2022). Analysis was guided by the research question and objectives. This approach enabled identification, analysis and reporting of themes in rich detail, allowing for complexities to be explored (Braun & Clarke, 2019). The authors (JARD, MA and GG) familiarised themselves with the included studies or data by reading and highlighting electronic or hard copies of each article. Initial codes were generated and then initial themes were constructed by listing, describing and comparing codes to examine relationships between codes and developing themes. Next, the authors reviewed the potential themes, defined and named the themes and produced a written report of the analysis (Braun & Clarke, 2022). This process was not linear; relationships between themes and subthemes were reconsidered throughout the process and on discussion with members of the research team. Table 3 shows examples of each step in this process. A thematic map and written report of the findings follow.

**TABLE 3:** Overview of thematic analysis with examples

**Step 1: Familiarisation with the data**
Two authors familiarised themselves with each study through reading, re‐reading and making initial notes. Sample notes included:
“Language barriers make it hard to identify what is a problem with health literacy, communication or confidence”; and “Women equated interpreters with understanding care, even though this did not appear to be true”.
**Step 2: Generating initial codes**
Each author generated initial codes systematically when rereading their allocated studies and reviewing initial notes. Codes included: “Lack of language resources”; and “Communication breakdown”.
**Step 3: Searching for themes**
Each author independently generated themes from the codes, collapsing codes which shared similar meanings or explored different aspects of the same concept. Initial themes included: “Barriers related to language” (coded by GG); “Language and logistical barriers” (coded by JARD); and “Limited English proficiency and issue with interpreters” (coded by MA).
**Step 4: Reviewing themes**
The authors shared preliminary themes which were compiled into a table to compare and contrast themes generated. These were reviewed in relation to the entire dataset (included studies) to generate a set of themes and sub‐themes. For example, the initial themes listed in Step 3 were compiled into the theme, “The language barrier”. Authors (JARD and MA) provided feedback on the themes and sub‐themes which were reviewed in relation to the dataset until consensus was achieved. For example, “The language barrier” was re‐positioned as a sub‐theme informing the broader intersectional factor or theme, “Language”.
**Step 5: Defining and naming themes**
The first author defined and named themes, producing a detailed analysis of themes and sub‐themes. A diagram was constructed to show how the themes were related.
**Step 6: Producing a written report**
The first author produced a written report of the themes. This was shared with all five authors for feedback and refined.

## 3 RESULTS

### 3.1 Description of included studies

All included studies were qualitative. Of these, seven studies solely explored the healthcare experiences of women of refugee background from Myanmar. The remaining studies included, in addition to women of refugee background from Myanmar, women of refugee background of Bhutanese/Nepali, Iraqi, Vietnamese, Assyrian, South Sudanese or Somali Bantu origin or men of refugee background from Myanmar. The majority of studies explored the healthcare experiences of women of Karen ethnicity (*n* = 11). Other ethnicities were reported as Chin (*n* = 2), Kachin (*n* = 2), Karenni (*n* = 2), Bamar/Burman (*n* = 2), Burmese (*n* = 1), Rohingya (*n* = 1) and unidentified (*n* = 3). The terms Bamar and Burman are synonymous with the word Burmese. Three studies included service provider perspectives along with those of women of refugee background from Myanmar. One study included community leaders, five of whom were women of refugee background from Myanmar. Studies were conducted in the United States (*n* = 8), Australia (*n* = 7) and Canada (*n* = 1).

The included studies explored a range of experiences accessing different types of healthcare. The majority explored aspects of healthcare unique to women (*n* = 9) such as birth, motherhood and sexual and reproductive health (SRH) including family planning and cervical cancer screening. The remaining experiences included mental health and wellbeing (*n* = 3), general health (*n* = 2), cancer screening (*n* = 1) and diabetes mellitus management (*n* = 1).

The majority of studies (*n* = 11) included clinician researchers, such as nurses or occupational therapists, amongst their authors (Clark, 2018; Jiwrajka et al., 2017; Kim et al., 2021; LaMancuso et al., 2016; Lenderts et al., 2021; Lor et al., 2018; Maung et al., 2021; Riggs et al., 2012; Schuster et al., 2019; Soin et al., 2020; Ussher et al., 2012). Two studies reported that a member of the research team were cultural insiders. One researcher reflected on her Chinese‐Burmese heritage when reporting on the positionality of the research team (Maung et al., 2021) and one researcher was a Karen woman of refugee background (Niner et al., 2014). Thirteen studies reported on the involvement of bicultural workers, key community informants or interpreters for participant recruitment, development of the data collection tool and data collection itself, including interpretation (Clark, 2018; Joseph et al., 2019; Joseph et al., 2020; Kumar, 2021; LaMancuso et al., 2016; Lenderts et al., 2021; Lor et al., 2018; Maung et al., 2021; Niner et al., 2013; Riggs et al., 2012; Schuster et al., 2019; Soin et al., 2020; Ussher et al., 2012).

**FIGURE 2:**
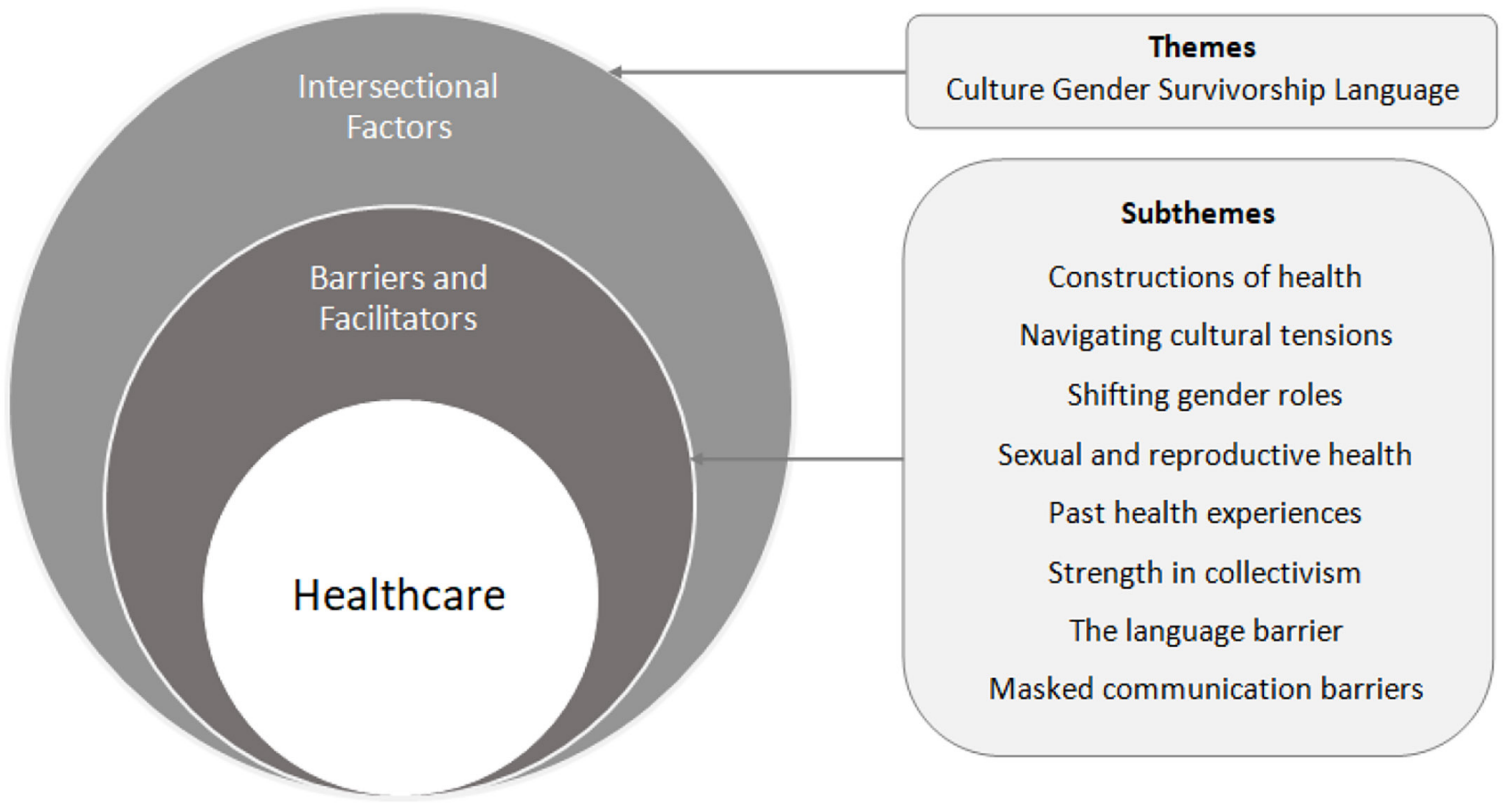
Themes diagram

We constructed four themes and eight subthemes during thematic analysis: Culture (Constructions of health; Navigating cultural tensions), Gender (Shifting gender roles; Sexual and reproductive health); Survivorship (Past health experiences; Strength in collectivism) and Language (The language barrier; Masked communication barriers). The four themes explored the intersectional factors which influenced the women's experiences of accessing and navigating healthcare. Subthemes explored the barriers and facilitators to women's healthcare access in the context of these overlapping factors. Figure 2 shows the relationship between the intersectional factors (themes), and barriers and facilitators to healthcare access (subthemes) identified.

### 3.2 Theme: Culture

Health was a cultural construction associated with traditional practices and concepts of wellbeing (Kumar, 2021; Lenderts et al., 2021). Culture shaped what being in good and poor health meant to women. Healthcare was the site of cultural tensions. Women attempted to navigate a foreign health system amidst these tensions.

#### 3.2.1 Subtheme: Constructions of health

Health was a culturally defined concept (Kumar, 2021; Lenderts et al., 2021). For women of refugee background from Myanmar, to be healthy was associated with being able to work and to actively contribute to the wellbeing of their families and community (Lenderts et al., 2021). Poor health was described as an inability to do or work, associated with stress and over‐thinking. These constructions of good and poor health influenced when women perceived a need to access healthcare (Lenderts et al., 2021). Aspects of Western healthcare such as primary and preventative health were typically unfamiliar concepts and as such, rarely sought out by women (Kumar, 2021; Lor et al., 2018; Schuster et al., 2019).

Mental health was highlighted as rich with cultural meanings by community leaders (Kim et al., 2021). This was reflected by Karen and Burmese mental illness terminology which could be associated with cultural stigma. Furthermore, symptoms of mental illness could be attributed to previous sins or evil spirits. Thus treatment may be sought from traditional practitioners instead of, or alongside Western biomedical treatments (Kim et al., 2021). For Karen women, postnatal depression was viewed as an alien experience due to the special cultural value placed upon motherhood (Ussher et al., 2012). This understanding of mental health and associated stigma was posited as a barrier to healthcare (Kim et al., 2021; Ussher et al., 2012).

#### 3.2.2 Subtheme: Navigating cultural tensions

When accessing healthcare, women navigated tensions between traditional and biomedical constructions of health (Joseph et al., 2019). Five studies reported on a misalignment between women's values and those of healthcare providers (Jiwrajka et al., 2017; Joseph et al., 2019; Joseph et al., 2020;LaMancuso et al., 2016; Niner et al., 2013). Two of these studies reported on the perspectives of healthcare providers (Jiwrajka et al., 2017; LaMancuso et al., 2016). While women trusted healthcare providers, they were perplexed by the Western biomedical approach to intrapartum care (LaMancuso et al., 2016; Niner et al., 2013). Women expressed gratitude for the safety of hospitals, however, they perceived conflict between their wishes and the medicalised birthing practices they experienced (LaMancuso et al., 2016). For women, birth and the postpartum period were reflected upon as a time of vulnerability, rich with cultural practices to recover and regain strength (Joseph et al., 2019; Joseph et al., 2020).

When women were not supported by healthcare providers to continue their traditional practices, they felt uncared for and their experiences of perinatal care were characterised negatively (Joseph et al., 2019). One American‐based study argued that the American healthcare system was simply not structured to meet the cultural and trauma‐informed health needs of people of refugee background from Myanmar (Kim et al., 2021). Short appointment durations, language barriers and perceived healthcare costs were presented as barriers to the provision of culturally safe healthcare (Jiwrajka et al., 2017; Joseph et al., 2019). A culture‐centred approach and cultural competency training for healthcare providers were recurrent recommendations from five studies (Jiwrajka et al., 2017; Joseph et al., 2019; Kim et al., 2021; Niner et al., 2014; Riggs et al., 2012).

### 3.3 Theme: Gender

Gender roles shifted on resettlement (Maung et al., 2021; Soin et al., 2020). Competing responsibilities could take priority over healthcare access; however, improved health knowledge could empower women (Clark, 2018;Niner et al., 2013; Soin et al., 2020). Cultural and gendered values, including stigmas, converged to shape women's perceptions of and access to sexual and reproductive healthcare (Ussher et al., 2012).

#### 3.3.1 Subtheme: Shifting gender roles

Shifting gender roles on resettlement could be a source of stress or empowerment for women (Maung et al., 2021; Soin et al., 2020). Maintaining good health was one of many priorities. On resettlement, women's responsibilities could include maintaining the household, caring for children, navigating resettlement challenges and providing financially for the family (Clark, 2018; Lenderts et al., 2021; Maung et al., 2021). These responsibilities left women time poor (Clark, 2018); they could be complicated by the loss of men's traditional status as provider, alcoholism and family violence (Maung et al., 2021; Soin et al., 2020). Co‐location of a refugee mentor at Karen playgroup was identified as a facilitating factor in Karen women's uptake of maternal and child health services (Riggs et al., 2012). The refugee mentor provided women with information about maternal and child health services while the women simultaneously met their gendered caregiving responsibilities.

Community leaders reported that resettlement challenges such as employment could take priority over health needs or directly influence access to healthcare (Kim et al., 2021). Alternatively, changed gender roles could be a source of empowerment (Niner et al., 2013; Soin et al., 2020). Younger Karen women had increased access to education and employment, fewer posttraumatic symptoms and more exposure to human rights discourse (Niner et al., 2013). This facilitated their sense of their healthcare entitlements; they were more likely to access healthcare and question health professionals (Soin et al., 2020).

#### 3.3.2 Subtheme: Sexual and reproductive health

Cultural values, including stigma, shaped women's sexual and reproductive health (SRH) knowledge and health service uptake (Lor et al., 2018; Soin et al., 2020; Ussher et al., 2012). Prior to marriage, silence and secrecy perpetuated a lack of SRH knowledge for young Karen women. Their use of SRH services was positioned as unacceptable (Ussher et al., 2012). Cultural modesty, embarrassment, fear and stigma were barriers to family planning services, abortion, cervical screening and discussing sexual pain with healthcare providers. Women preferred healthcare providers to initiate SRH conversations and a female healthcare provider (Lor et al., 2018; Soin et al., 2020; Ussher et al., 2012).

Amongst married women, health knowledge and literacy were highest in relation to fertility, reflecting the cultural value placed on motherhood (Ussher et al., 2012). Women gained some knowledge mixed with misinformation about contraception and sexually transmitted diseases in refugee camps (Soin et al., 2020). Resettlement in the United States was associated with improved family planning knowledge. This, combined with increased socioeconomic opportunities, empowered Burmese women to make their own reproductive decisions (Soin et al., 2020). Conversely, on resettlement in Australia, Karen women's husbands continued to be the contraceptive decision‐makers despite women's improved contraceptive knowledge (Ussher et al., 2012).

### 3.4 Theme: Survivorship

Health was viewed within the context of survivorship, including survivorship of past threats to their health (Kumar, 2021). These experiences influenced women's health knowledge and expectations of healthcare (Kumar, 2021; LaMancuso et al., 2016; Lor et al., 2018). Collectivism emerged as a culturally influenced response to trauma and facilitated healthcare access (Clark, 2018; Kumar, 2021; Lenderts et al., 2021;Lor et al., 2018; Maung et al., 2021).

#### 3.4.1 Subtheme: Past health experiences

Past experiences of health and healthcare influenced women's health knowledge and expectations of healthcare on resettlement. These past experiences were framed within the context of survival and past trauma in three studies (Kumar, 2021; LaMancuso et al., 2016; Lor et al., 2018). Healthcare was also typically used in emergency situations in refugee camps and thus, was reserved for emergency use on resettlement (Schuster et al., 2019). Treatments for manageable conditions such as diabetes may have been unavailable in refugee camps exposing women to the life‐threatening effects of these conditions (Kumar, 2021; Schuster et al., 2019). One study reported, for example, that Karen women held a fatalistic view of cancer because in the refugee camps, cancer was typically diagnosed at an advanced stage and curative treatment was not available (Schuster et al., 2019). This fatalism impeded women's engagement with screening services on resettlement.

#### 3.4.2 Subtheme: Strength in collectivism

Women drew upon a collective culture to overcome barriers to healthcare (Clark, 2018; Maung et al., Kumar, 2021; Lenderts et al., 2021; Lor et al., 2018). Five studies identified women utilising community networks on resettlement to navigate health needs, improve health knowledge and access healthcare (Clark, 2018; Maung et al., Kumar, 2021; Lenderts et al., 2021; Lor et al., 2018). One of these studies included community leaders in the sampled population (Clark, 2018). Women asked community members and their own children to translate for them, enabling them to make appointments or communicate with healthcare providers (Clark, 2018; Lenderts et al., 2021; Lor et al., 2018).

Women of refugee background from Myanmar valued being able to assist each other with their health needs and share their problems (Lor et al., 2018; Maung et al., 2021). They viewed themselves as survivors and took pride in assisting those in need (Maung et al., 2021). When women had resettled in locations where there was not a pre‐existing migrant community from Myanmar, they utilised religious groups and settlement services to facilitate their healthcare access and navigate resettlement challenges (Maung et al., 2021). Women preferred to receive support from other migrants from Myanmar. However, some women felt that they were made dependent on such community figures (Clark, 2018).

Four studies posited collectivism as a strength which could be utilised to improve healthcare access (Kumar, 2021; Lenderts et al., 2021; Lor et al., 2018; Maung et al., 2021). Karen doulas were identified as a successful example of formal peer roles by Karen interpreters and Karen doulas themselves; they interpreted, assisting women to overcome language barriers, supported Karen women to navigate perinatal health services and advocated for women (LaMancuso et al., 2016). Karen refugee mentors were another example of a formal bicultural peer role; they facilitated access to maternal and child health services (Riggs et al., 2012).

### 3.5 Theme: Language

Language could inhibit or facilitate women's access to and navigation of healthcare (Clark, 2018; Jiwrajka et al., 2017; Joseph et al., 2019; Kim et al., 2021; Kumar, 2021; LaMancuso et al., 2016; Lenderts et al., 2021; Lor et al., 2018; Maung et al., 2021; Niner et al., 2013; Niner et al., 2014; Riggs et al., 2012; Schuster et al., 2019; Soin et al., 2020). The presence of an interpreter was typically equated with effective communication; however, self‐effacement and inadequate health literacy also contributed to communication barriers (Clark, 2018; Jiwrajka et al., 2017; Kumar, 2021; LaMancuso et al., 2016; Maung et al., 2021; Niner et al., 2013; Riggs et al., 2012; Ussher et al., 2012).

#### 3.5.1 Subtheme: The language barrier

Language barriers were reported in 14 of 16 included studies (Clark, 2018; Jiwrajka et al., 2017; Joseph et al., 2019; Kim et al., 2021; Kumar, 2021; LaMancuso et al., 2016; Lenderts et al., 2021; Lor et al., 2018; Maung et al., 2021; Niner et al., 2013; Niner et al., 2014; Riggs et al., 2012; Schuster et al., 2019; Soin et al., 2020). Where language barriers arose, communication could be impeded. Language barriers could persist despite interpreter use (Jiwrajka et al., 2017; Lor et al., 2018; Soin et al., 2020). Problems associated with interpreter use included language or dialect differences between the woman and the interpreter (Jiwrajka et al., 2017; Lor et al., 2018), healthcare professionals inadequately trained to work with interpreters (Jiwrajka et al., 2017), the use of telephone interpreters (Riggs et al., 2012) and difficulty translating concepts across languages (Kim et al., 2021). A lack of interpreters or healthcare providers not offering interpreters were also cited as common problems (Niner et al., 2014; Riggs et al., 2012). Continuity of interpreter, in‐person interpreter‐mediated appointments and translation of written information were recommended (Niner et al., 2014; Riggs et al., 2012). Bilingual peer roles such as Karen doulas or refugee mentors also assisted women to overcome language barriers (LaMancuso et al., 2016; ).

#### 3.5.2 Subtheme: Masked communication barriers

When communication was identified as ineffective, language barriers were highlighted (Jiwrajka et al., 2017). However, language was not the only barrier to effective communication which impeded women's access to and navigation of healthcare. Health literacy was identified as a factor in healthcare access or health knowledge in five studies (Clark, 2018; Jiwrajka et al., 2017; Kumar, 2021; Riggs et al., 2012; Ussher et al., 2012). Women were challenged by communicating and understanding information about health, making appointments or locating health services (Clark, 2018). Essentially women were challenged by functional aspects of health literacy in a foreign healthcare system (Clark, 2018). Language barriers, a reluctance to ask questions, conflicting cultural values, fear, perceived discrimination and unfriendly body language from healthcare providers all contributed to women's difficulty navigating healthcare (LaMancuso et al., 2016; Niner et al., 2013). Conversely, positive relationships with healthcare providers, continuity of healthcare provider and Karen doulas could facilitate women's navigation of health services (LaMancuso et al., 2016; Riggs et al., 2012). Concepts of self‐effacement, agreeability, shyness or “gracious acceptance” (Niner et al., 2013, p541) were identified in four studies as preventing women from asking questions and advocating for themselves (Clark, 2018; LaMancuso et al., 2016; Maung et al., 2021; Niner et al., 2013). Authors reported that women of refugee background from Myanmar demonstrated a reluctance to complain and a predisposition towards self‐effacement despite negative healthcare experiences (Clark, 2018; LaMancuso et al., 2016; Maung et al., 2021; Niner et al., 2013). The term self‐effacement referred to women seeking not to draw attention to themselves and to display humility and modesty (Maung et al., 2021). Karen women often conveyed gratitude and acceptance despite being dissatisfied or not understanding the care they received (LaMancuso et al., 2016). This behaviour was attributed to low self‐efficacy, previous experiences of trauma and cultural and gendered expectations. Karen women, however, perceived themselves as self‐reliant and adept at controlling their emotions; they did not want to ask for help and be viewed as dependent (Clark, 2018; Niner et al., 2014). Trained in perinatal care, Karen doulas were confident to advocate for these women and encouraged the women to advocate for themselves (LaMancuso et al., 2016).

## 4 DISCUSSION

Our findings offer novel insight into the healthcare experiences of women of refugee background from Myanmar on resettlement in signatory countries. Thematic analysis generated four themes reflecting the four factors which overlapped to shape women's experiences of accessing and navigating healthcare. Class, culture and race or ethnicity have previously been identified as important factors in health inequalities (Kim et al., 2021). This review found that for women of refugee background from Myanmar, survivorship and language, along with traditional factors of gender and culture, interacted to shape the barriers women faced accessing and navigating healthcare, and the facilitators which they drew upon to overcome barriers. This valuable insight can be used to co‐design or modify community health interventions and healthcare provider training. This discussion will explore potential interventions which emerged from the findings, culturally responsive training, community education and bicultural health navigators.

### 4.1 Culturally responsive trauma‐informed training

Our findings revealed that healthcare was navigated amidst cultural tensions, shifting gender roles, prior trauma and communication barriers. Western healthcare systems were identified as poorly structured to meet the cultural and trauma‐informed needs of people of refugee background from Myanmar due to systemic factors such as short appointment times and difficulty accessing interpreters (Kim et al., 2021). Language barriers, health literacy and self‐effacing tendencies were shown in this scoping review to impede effective communication and thus, healthcare access and navigation.

Cultural competency training for healthcare providers was a recurrent recommendation to support healthcare providers to improve their skills and confidence when caring for women of refugee background from Myanmar (Jiwrajka et al., 2017; Joseph et al., 2019; Kim et al., 2021; Niner et al., 2014; Riggs et al., 2012). Cultural competence training or education has been widely recommended to improve healthcare providers' capacity to meet the health needs of people of refugee and asylum‐seeker background (Lau & Rodgers, 2021). Such training is designed to enhance the provision of patient‐centred care by educating clinicians to value diversity, integrate cultural knowledge, assess cross‐cultural interactions and partake in reflective practice (Watt et al., 2015). However, criticism of cultural competence training has included stereotyping, emphasis of cultural differences and reliance on patients to express their cultural needs. As this scoping review highlights, women of refugee background from Myanmar may demonstrate self‐effacement and reluctance to initiate questions on culturally stigmatised subjects such as SRH.

Alternatively to cultural competence training programs, culturally responsive trauma‐informed training workshops have been trialled with mental healthcare providers and leaders of refugee background communities (Im & Swan, 2021). In contrast to cultural competence training programs, this community‐based participatory approach is centred on learning from people of refugee and asylum seeker backgrounds (Im & Swan, 2021; Lau & Rodgers, 2021). Community leaders were essential to the co‐design and delivery of these workshops, the purpose of which was to facilitate intercultural learning and nurture trust and relationships between community leaders and healthcare providers. The workshops focused on cultural strengths and barriers to healthcare, including cultural interpretations of mental health. This scoping review revealed that women of refugee background from Myanmar strongly identified as survivors of trauma. Culturally responsive trauma‐informed training co‐designed and delivered in partnership with community leaders may assist healthcare providers who care for women of refugee background from Myanmar to improve their cultural competency while enhancing community relationships.

### 4.2 Community education programs

For women of refugee background from Myanmar, health is a cultural construction. In their ethnographic study of people of refugee background from Bhutan resettled in the United States, Chao and Kang (2020) reported that health literacy is also a socio‐cultural concept, mediated by tradition, language and experiences and associated with community engagement. Similarly, this scoping review revealed that women of refugee background from Myanmar drew upon community resources to facilitate their healthcare access and navigation. Collectivism emerged as a salutogenic factor that may be enhanced to facilitate improved access to and navigation of healthcare. Salutogenic factors may be used to maintain or improve health and wellbeing. Collectivism has been recognised as a salutogenic factor in previous research on communities of refugee origin including those from Myanmar (Chao & Kang, 2020; Wong et al., 2020). Community education programs have been recommended to address misinformation, improve community health knowledge and health literacy, and address cultural stigmas (Chao & Kang, 2020; Frost, 2016; Kim et al., 2021; Schuster et al., 2019).

Community education programs can develop community capacity, facilitate social networks and peer‐to‐peer learning (Frost, 2016). A community health education program was trialled with women of refugee background from Myanmar resettled in the United States (Frost, 2016). This program included workshops, excursions and home visits. Health educators of refugee background were supported with training to co‐facilitate the program; they were essential to recognise cultural and community nuances. Education materials were developed in consideration of language and literacy needs. Frost (2016) reported that women were more likely to engage with the program if it also addressed competing resettlement priorities such as development of English language skills.

### 4.3 Bicultural health navigators

A key finding of this scoping review is that bilingual peers formally and informally facilitated healthcare access and navigation for women of refugee background from Myanmar. Karen doulas and refugee mentors assisted women to overcome language barriers, navigated cultural tensions, advocated for women and encouraged the development of their healthcare knowledge and health literacy by sharing knowledge and encouraging them to ask questions. Formal bicultural peer roles have been trialled in several healthcare settings (Henderson & Kendall, 2011; Wei et al., 2021). There is significant variation in how the role is defined, named and implemented; examples include bicultural workers, refugee liaisons and multicultural health brokers (Wei et al., 2021).

The role typically extends beyond that of interpreters. They may act as a resource of cultural and community knowledge for service providers, and of healthcare knowledge for community members. An Australian study reported on the role of community navigators working with refugee‐background communities, including the Burmese community (Henderson & Kendall, 2011). Community navigators were bilingual individuals who were already informal community health brokers or leaders and undertook special training. They assessed the needs of their clients, facilitated health promotion and healthcare access, supported healthcare providers to work with interpreters and made appropriate referrals. Essentially, community navigators acted as knowledge brokers for members of their community and healthcare providers. However, they often worked beyond their established working hours and were at high risk for burnout.

Bicultural health navigators have the potential to improve health literacy skills and health knowledge for women of refugee background from Myanmar. This scoping review showed that Karen doulas and refugee mentors had unique insight into the needs of their peers, acting as cultural health brokers, interpreting and assisting women to navigate barriers to healthcare and to understand their healthcare entitlements (LaMancuso et al., 2016; Riggs et al., 2012). Similarly, Chao and Kang (2020) observed that people of refugee background from Bhutan resettled in the United States, engaged formal and informal cultural and literacy brokers to bridge language, cultural and health literacy barriers with healthcare providers. Formalisation of such roles may facilitate improved communication and thus, improve women's ability to navigate and access healthcare. Further research is needed into how this role may be established as a sustainable intervention, and the short and long‐term impacts it may have on health outcomes and health literacy.

### 4.4 Strengths and Limitations

Scoping review methodology facilitates the synthesis of a range of evidence, offering insight into a topic on which there is little evidence. The present study offered, for the first time, novel insight into the healthcare experiences of women of refugee background from Myanmar on resettlement. However, as the review was limited to only the experiences of women of refugee background from Myanmar, the findings are of limited generalisability to other populations. Furthermore, the majority of the studies reported on explored the experiences of Karen women, therefore, further research into the specific experiences of women of Karenni, Mon or other ethnicities from Myanmar is recommended. Additionally, as only Anglophone countries were identified in this review, the findings are of limited generalisability for non‐English speaking countries such as South Korea. This reflects the decision to exclude non‐English language articles from this scoping review. A Critical Appraisal Skills Programme checklist was used to assess each source for rigour (Critical Appraisal Skills Programme, 2019). No sources were identified for exclusion during this process.

Although the majority of studies included cultural insiders in their participant recruitment and data collection processes (*n* = 13), a minority of studies included people from Myanmar or of Myanmar heritage as authors or reported on their involvement in data analysis (*n* = 2). Bicultural researchers or research assistants contribute key insider knowledge about a community and may share lived experiences with participants (Ragavan & Cowden, 2020). These attributes can facilitate engagement with participants and may result in disclosure of experiences they would not otherwise share. However, exclusion of bicultural researchers from analysis should be considered a methodological limitation as the findings do not benefit from the bicultural researchers' insider cultural knowledge and may result in the misinterpretation of cultural nuance. This limitation of the sources themselves may also be considered a limitation of this scoping review.

## 5 CONCLUSION

This scoping review revealed that the healthcare experiences of women of refugee background from Myanmar were located at the intersection of culture, gender, survivorship and language. A number of barriers to healthcare for these women were identified. A major barrier was language, however, health literacy and self‐effacing tendencies also impeded communication with healthcare providers. Further research into the role of these elements is recommended. Culturally responsive trauma‐informed training programs are offered as a potential intervention to support healthcare providers to better care for this community of women. Finally, community networks and bilingual peers were identified as salutogenic resources that women draw upon to facilitate their access to and navigation of healthcare. Further research is recommended into interventions which may build upon pre‐existing community resources to improve community health outcomes, such as community education programs and bicultural health navigators. Ultimately, understanding the barriers and facilitators to healthcare experienced by women of refugee background from Myanmar, and the intersectional influences on their health, is central to tailoring services, education and interventions to their needs.

## AUTHORS' CONTRIBUTIONS

All authors contributed to the study conception and design. Data collection was performed by GG. Data analysis was performed by GG, MA and JARD. The first draft of the manuscript was written by GG. All authors contributed to revisions and edits of the manuscript. All authors read and approved the final manuscript.

## Funding information

The authors declare that no funds or grants were received during the preparation of this manuscript.

## CONFLICT OF INTEREST

They have no relevant or non‐financial interests to disclose, or competing interests to declare relevant to the content of this article.

## ETHICS APPROVAL

The first author (GG) is a Master of Philosophy student at Curtin University and this scoping review is part of her thesis. As such, ethics approval was obtained from the Curtin University Human Research Ethics Committee (HRE2020‐0721). GG is supported by the RTP (Research Training Program) scheme of the Australian government.

## Data Availability

The datasets generated during and/or analysed during the current study are available from the corresponding author on reasonable request.
